# Is the Complement Protein C1q a Pro- or Anti-tumorigenic Factor? Bioinformatics Analysis Involving Human Carcinomas

**DOI:** 10.3389/fimmu.2019.00865

**Published:** 2019-05-03

**Authors:** Alessandro Mangogna, Chiara Agostinis, Deborah Bonazza, Beatrice Belmonte, Paola Zacchi, Gabriella Zito, Andrea Romano, Fabrizio Zanconati, Giuseppe Ricci, Uday Kishore, Roberta Bulla

**Affiliations:** ^1^Department of Life Sciences, University of Trieste, Trieste, Italy; ^2^Institute for Maternal and Child Health, Istituto di Ricovero e Cura a Carattere Scientifico (IRCCS) Burlo Garofolo, Trieste, Italy; ^3^Department of Medical, Surgical and Health Science, University of Trieste, Trieste, Italy; ^4^Tumor Immunology Unit, Human Pathology Section, Department of Health Sciences, University of Palermo, Palermo, Italy; ^5^Biosciences, College of Health and Life Sciences, Brunel University London, Uxbridge, United Kingdom

**Keywords:** complement, classical pathway, C1q, tumor, microenvironment, prognosis

## Abstract

C1q is the first subcomponent of the classical pathway of the complement system and belongs to the C1q/Tumor Necrosis Factor superfamily. C1q can perform a diverse range of immune and non-immune functions in a complement-dependent as well as -independent manner. Being a pattern recognition molecule of the innate immunity, C1q can recognize a number of self, non-self and altered-self ligands and bring about effector mechanisms designed to clear pathogens via opsonisation and inflammatory response. C1q is locally synthesized by macrophages and dendritic cells, and thus, can get involved in a range of biological processes, such as angiogenesis and tissue remodeling, immune modulation, and immunologic tolerance. The notion of C1q involvement in the pathogenesis of cancer is still evolving. C1q appears to have a dual role in cancer: tumor promoting as well as tumor-protective, depending on the context of the disease. In the current study, we performed a bioinformatics analysis to investigate whether C1q can serve as a potential prognostic marker for human carcinoma. We used the Oncomine database and the survival analysis platforms Kaplan-Meier plotter. Our results showed that high levels of C1q have a favorable prognostic index in basal-like breast cancer for disease-free survival, and in HER2-positive breast cancer for overall survival, while it showed a pro-tumorigenic role of C1q in lung adenocarcinoma, and in clear cell renal cell carcinoma. This *in silico* study, if validated via a retrospective study, can be a step forward in establishing C1q as a new tool as a prognostic biomarker for various carcinoma.

**Graphical Abstract:**
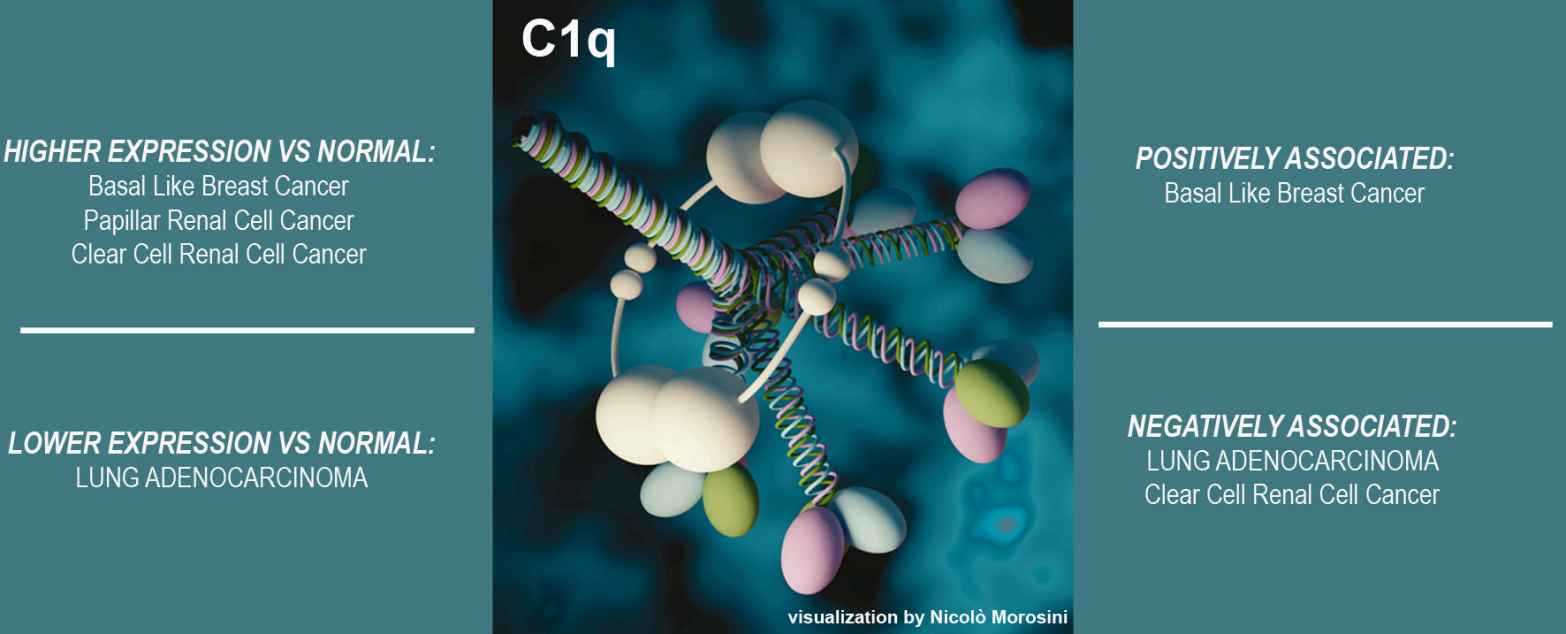
Summary of the conclusions of the study.

## Introduction

C1q is the first recognition subcomponent of the complement classical pathway, which when associated with C1r and C1s, forms a C1 complex, allowing the activation of the complement cascade (1). By virtue of its ability to bind to IgG and IgM containing immune complexes and activating the classical pathway, C1q acts as prototypical link between innate and adaptive immune wings of the immune system (2). C1q can bind to a range of non-self-target ligands (pathogens), altered self (β-amyloid peptide, prion protein, apoptotic and necrotic cells via phosphatidylserine and DNA, respectively), and cell surface receptors (such as calreticulin and gC1qR) (3). Several features of the C1q render it a versatile molecular sensor of damage-modified self or non-self antigens (4). C1q, unlike most of the complement proteins which are exclusively produced by hepatocytes, can also be synthesized in a local environment by a wide range of cell types including macrophages and dendritic cells (5). Local synthesis, therefore, offers an additional avenue to C1q in order to exert specific functions *in situ* that are strictly connected to its site of production without involving complement activation (6).

C1q is an hexametric glycoprotein of about 460 kDa, resembling a “bouquet of tulips” being composed by three polypeptide chains: A (28 kDa), B (25 kDa), C (24 kDa), which are the product of three distinct genes clustered in the same orientation, and in the order A–C–B, on a 24 kb stretch of DNA on chromosome 1p (7). Each chain consists of a C-terminal globular head (gC1q) domain and an N-terminal triple-helical collagen-like (cC1q) domain (8). C1q associates with the Ca^2+^-dependent C1r_2_-C1s_2_ tetramer, of about 360 kDa, to form the soluble pentameric C1 complex (9). The C-terminal ends of A, B and C chains assemble together to form a heterotrimeric gC1q domain, which by virtue of its modular organization, can work independently and engage with a diverse range of target ligands (3). While the gC1q domain latches on to the charge patterns on the ligands, the cC1q domain can interact with effector mechanism inducers, such as C1r, C1s, cell surface receptors, etc. Thus, a combination of a highly versatile and modular gC1q domain and a cell surface interacting cC1q domain, together with its local synthesis, makes C1q a potent orchestrator of molecular pathways. C1q is involved not only in innate and adaptive immune mechanisms, but also in a wide range of physiological and pathological processes, such as placental development (10, 11), pre-eclampsia (12, 13), wound healing (14) and cancer (15–18).

Markiewski et al. provided evidence that C1q is present in syngeneic mouse tumors. Indeed, they found that the activation of the classical pathway is the major contributor to complement-mediated tumor progression (19). Subsequently, we showed that locally expressed C1q had important effects in the tumor microenvironment (TME) (17). C1q expressed in the stroma and vascular endothelium of several human malignant tumors acted as a tumor-promoting factor by favoring adhesion, migration and proliferation of cancer cells as well as angiogenesis and metastasis. C1q-deficient (C1qa^−/−^) mice, bearing a syngeneic B16 melanoma, exhibited slower tumor growth and prolonged survival, compared to C3 or C5 deficient mice although it has been shown that C3/C5 deficiency may also create microenvironment suboptimal for tumor growth (20, 21). Recently, we demonstrated that C1q is abundantly present in malignant pleural mesothelioma (MPM), where it can combine with hyaluronic acid (HA), which is a principal component of the TME, and enhance the tumor growth by promoting cell adhesion and proliferation (18). However, other have shown a pro-apoptotic effect of C1q on prostate (15) and ovarian cancer cells *in vitro* (16). These rather two set of contradicting studies warranted a systematic analysis of the context of the disease and TME that can render C1q protective or pathogenic in cancer.

In the current study, we performed a bioinformatics analysis, using Oncomine database and the survival analysis platforms Kaplan-Meier plotter, in order to investigate whether C1q can serve as a potential prognostic marker for human carcinoma, i.e., tumors of epithelial origin. Our results showed that high levels of C1q have a favorable prognostic index in basal-like breast cancer (BLBC) and in HER-2 positive breast cancer. However, we found a pro-tumorigenic role of C1q in lung adenocarcinoma, and in clear cell renal cell carcinoma (CCRCC). This study is an important step forward in highlighting C1q as a new prognostic candidate biomarker for a range of carcinomas.

## Methods

### Oncomine Database Analysis

The expression levels of *C1QA, C1QB*, and *C1QC* genes in various carcinomas were analyzed using Oncomine (www.oncomine.org), a cancer microarray database and web-based data mining platform from genome-wide expression analyses (22, 23). We compared the differences in mRNA level between normal tissue and carcinoma. The mRNA expression levels in neoplastic tissues compared to the healthy tissues were obtained as the parameters of *p*-value < 0.05, fold change >2, and gene ranking in the top 10%. Information about the dataset used in this study is summarized in Supplementary Table 1.

### Kaplan-Meier Plotter Database Analysis

A Kaplan-Meier plotter database can be used to assess the effect of 54,675 genes on survival using 10,461 carcinoma samples (5,143 breast, 1,816 ovarian, 2,437 lung, and 1,065 gastric cancer patients with a mean follow-up of 69/40/49/33 months) using probe sets on the HGU133 Plus 2.0 array from Gene Expression Omnibus (GEO). For other human carcinoma, a total of 3,439 patients with RNA HiSeq data from The Cancer Genome Atlas (TCGA) cohort were collected. The prognostic significance of *C1QA, C1QB*, and *C1QC* expression and survival in several carcinomas was analyzed by Kaplan-Meier plotter (www.kmplot.com/analysis/) (24). The hazard ratio with 95% confidence intervals and logrank *p*-value was also computed.

### Immunohistochemical Analysis

Normal and neoplastic human tissues, including breast, kidney and lung, were selected from the archives of the Department of Pathology, University of Trieste. Immunohistochemistry (IHC) was performed using a polymer detection method. Briefly, tissue samples were fixed in 10% v/v buffered formalin and then paraffin embedded. Four μm-thick tissue sections were deparaffinized and rehydrated. The antigen unmasking technique was carried out using Novocastra Epitope Retrieval Solutions, pH 9 (Leica Biosystems) in a PT Link pre-treatment module (Dako) at 98°C for 30 min. Sections were then brought to RT and washed in PBS. After neutralization of the endogenous peroxidase with 3% v/v H_2_O_2_ and Fc blocking by a specific protein block (Novocastra, Leica Biosystems), samples were incubated overnight at 4°C with rabbit polyclonal anti-human C1q (dilution 1:200) antibodies (Dako). Staining was carried out via polymer detection kit (Novocastra, Leica Biosystems) and DAB (3,3′-Diaminobenzidine; Dako, Denmark) substrate-chromogen. Slides were counterstained with Harris Haematoxylin (Novocastra, Leica Biosystems). Sections were analyzed under the Axio Scope A1 optical microscope (Zeiss) and microphotographs were collected through the Axiocam 503 color digital camera (Zeiss) using the Zen2 software.

### Statistical Analysis

Survival curves were generated by the Kaplan-Meier plotter. All results are displayed with *p*-values from a log-rank test. *P*-values < 0.05 were considered significant. Similarly, with Oncomine, the statistical significance of data (*p*-values) was provided by the program.

## Results

### Bioinformatic Analysis of the Three Genes Encoding Human C1q A, B, and C Chains in Normal Epithelial Tissues and Carcinomas

The expression of *C1QA, C1QB*, and *C1QC* genes was analyzed between different carcinoma and normal tissue counterparts using the Oncomine database. The threshold was determined as the following values: *p*-value < 0.05, fold change >2, and gene ranking in the top 10%. Carcinomas included in this analysis were: bladder carcinoma, breast cancer, cervical squamous cell carcinoma, esophageal carcinoma, head-neck squamous cell carcinoma, clear cell renal cell carcinoma (CCRCC), papillary renal cell carcinoma (PRCC), liver hepatocellular carcinoma, lung adenocarcinoma, lung squamous cell carcinoma, ovarian cancer, pancreatic ductal adenocarcinoma, rectum adenocarcinoma, gastric carcinoma, and uterine corpus endometrial carcinoma. We only investigated carcinomas in which all the three C1q chains showed a significant prognostic effect by Kaplan-Meier plotter analysis. The *C1QA, C1QB*, and *C1QC* genes were either over-expressed, or downregulated depending on the type of carcinoma investigated, as compared to their normal tissue counterparts. All the three C1q chains showed a differential prognostic significance. These data appear to suggest that C1q can have pro- or anti-tumorigenic implications, depending on the carcinoma types (Table 1). Thus, detailed analyses of the expression profiles of all three C1q chains were performed.

**Table 1:** Prognostic significance of C1q in patients with carcinomas.

**CANCER**	**Cancer subtype**	**Gene symbol**	**DFS/PFS**	**OS**
	***n* = number of patients**			
Breast^a^	Triple-negative	C1QA	HR = 0.47 (0.34–0.66)	HR = 0.52 (0.32–0.85)
	*n* = 618 for DFS		*p*-value (4.7e-6)	*p*-value (0.0079)
	*n* = 241 for OS			
Breast^a^	Triple-negative	C1QB	HR = 0.56 (0.43–0.72)	HR = 0.46 (0.28–0.75)
	*n* = 618 for DFS		*p*-value (5.6e-6)	*p*-value (0.0014)
	*n* = 241 for OS			
Breast^a^	Triple-negative	C1QC	HR = 0.58 (0.42–0.8)	HR = 0.38 (0.2–0.71)
	*n* = 360 for DFS		*p*-value (0.0009)	*p*-value (0.0019)
	*n* = 153 for OS			
Breast^a^	Luminal A	C1QA	HR = 1.31 (1.1–1.55)	ns
	*n* = 1,933 for DFS		*p*-value (0.0021)	
	*n* = 611 for OS			
Breast^a^	Luminal A	C1QB	HR = 1.54 (1.29–1.83)	HR = 2.09 (1.47–2.97)
	*n* = 1933 for DFS		*p*-value (1.0e-6)	*p*-value (2.6e-5)
	*n* = 611 for OS			
Breast^a^	Luminal A	C1QC	HR = 1.36 (1.07–1.74)	ns
	*n* = 841 for DFS		*p*-value (0.0132)	
	*n* = 271 for OS			
Breast^a^	Luminal B	C1QA	ns	ns
	*n* = 1,149 for DFS			
	*n* = 433 for OS			
Breast^a^	Luminal B	C1QB	ns	ns
	*n* = 1149 for DFS			
	*n* = 433 for OS			
Breast^a^	Luminal B	C1QC	ns	ns
	*n* = 407 for DFS			
	*n* = 129 for OS			
Breast^a^	HER2+	C1QA	HR = 0.49 (0.33–0.72)	HR = 0.17 (0.08–0.39)
	*n* = 251 for DFS		*p*-value (0.0002)	*p*-value (2.1e-6)
	*n* = 117 for OS			
Breast^a^	HER2+	C1QB	HR = 0.61 (0.37–0.99)	HR = 0.26 (0.12–0.55)
	*n* = 251 for DFS		*p*-value (0.0434)	*p*-value (0.0001)
	*n* = 117 for OS			
Breast^a^	HER2+	C1QC	ns	HR = 0.28 (0.13–0.63)
	*n* = 156 for DFS			*p*-value (0.001)
	*n* = 73 for OS			
Kidney^b^	Clear cell renal cell carcinoma	C1QA		HR = 1.76 (1.3–2.38)
	*n* = 530 for OS			*p*-value (0.0002)
Kidney^b^	Clear cell renal cell carcinoma	C1QB		HR = 1.55 (1.15–2.1)
	*n* = 530 for OS			*p*-value (0.0035)
Kidney^b^	Clear cell renal cell carcinoma	C1QC		H = 1.65 (1.21–2.24)
	*n* = 530 for OS			*p*-value (0.0012)
Kidney^b^	Papillary renal cell carcinoma	C1QA		ns
	*n* = 287 for OS			
Kidney^b^	Papillary renal cell carcinoma	C1QB		ns
	*n* = 287 for OS			
Kidney^b^	Papillary renal cell carcinoma	C1QC		ns
	*n* = 287 for OS			
Lung^c^	Adenocarcinoma	C1QA		HR = 2.11 (1.66–2.68)
	*n* = 720 for OS			*p*-value (6.4e-10)
Lung^c^	Adenocarcinoma	C1QB		HR = 1.83 (1.45–2.31)
	*n* = 720 for OS			*p*-value (2.0e-7)
Lung^c^	Adenocarcinoma	C1QC		HR = 3.29 (2.39–4.52)
	*n* = 673 for OS			*p*-value (9.9e-15)
Lung^c^	Squamous cell carcinoma	C1QA		ns
	*n* = 524 for OS			
Lung^c^	Squamous cell carcinoma	C1QB		ns
	*n* = 524 for OS			
Lung^c^	Squamous cell carcinoma	C1QC		HR = 0.64 (0.46–0.89)
	*n* = 271 for OS			*p*-value (0.0084)

a *Using 5,143 cancer samples on the HGU133 Plus 2.0 array from Gene Expression Omnibus, GEO*.

b *Using 817 cancer samples on the RNA HiSeq data from The Cancer Genome Atlas, TCGA*.

c *Using 2,437 cancer samples on the HGU133 Plus 2.0 array from GEO*.

*DSF, disease-free survival; OS, overall survival*.

### Significance of C1q Expression in Breast Carcinoma

**Figure 1:**
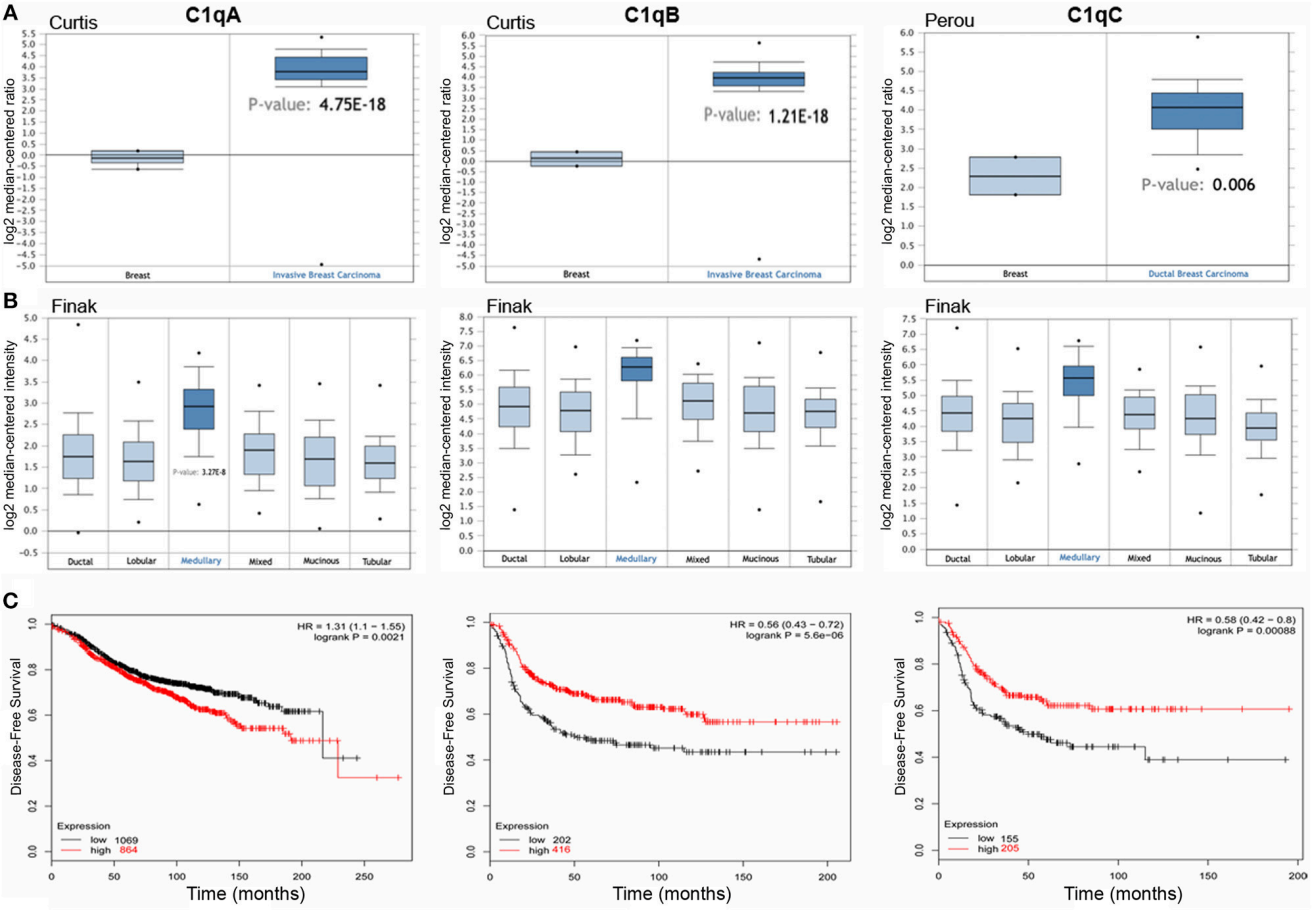
*C1QA, C1QB*, and *C1QC* expression in invasive breast carcinoma. Curtis's datasets were used for bioinformatics analysis to explore *C1QA* and *C1QB* mRNAs expression in the breast cancer, whereas Perou's datasets was used for bioinformatics analysis to evaluate *C1QC* mRNA. A higher C1q mRNAs expression was detectable in invasive breast carcinoma compared to normal breast tissue **(A)**. The analysis of the different breast carcinoma histotypes by Finak's dataset revealed how the medullary breast cancer presented the major intensity of C1q mRNA expression **(B)**. According to the data Kaplan-Meir plotter, *C1QA, C1QB*, and *C1QC* mRNA expressions was positively linked to a disease-free survival (DFS) rate in patients with basal-like cancers **(C)** (*p*<0.05) and to an overall survival (OS) rate with HER-2 positive cancers (Supplementary Table 2). HR, hazard ratio.

Bioinformatics analysis of *C1QA, C1QB*, and *C1QC* mRNA expression was performed in the context of the breast cancer using Karnoub's, Finak's, Curtis's, and Perou's datasets. A higher expression level of the three chains of C1q was detected as compared to normal breast tissue (Figure 1A, *p* < 0.05). When breast cancer was stratified into different histological subtypes, *C1QA, C1QB*, and *C1QC* mRNA expression achieved a statistical significance only in medullary carcinoma (Figure 1B, *p* < 0.05). To evaluate the prognostic significance of C1q in all breast cancers, we considered their molecular classification, such as luminal-A, luminal-B, HER-2 positive, and basal-like cancers (BLBC) (Supplementary Table 2).

According to Kaplan-Meir plotter data, *C1QA, C1QB*, and *C1QC* mRNA expression was positively associated with a disease-free survival (DFS) rate in patients with BLBC (Figure 1C, *p* < 0.05) and with an overall survival (OS) rate with HER-2 positive cancers (Table 1). This correlation was not evident in luminal-A and luminal-B patients. Only *C1QB* mRNA expression was negatively associated with high DFS and OS rates in the breast cancer patients with luminal-A, and to a DFS rate with all breast cancer.

**Figure 2:**
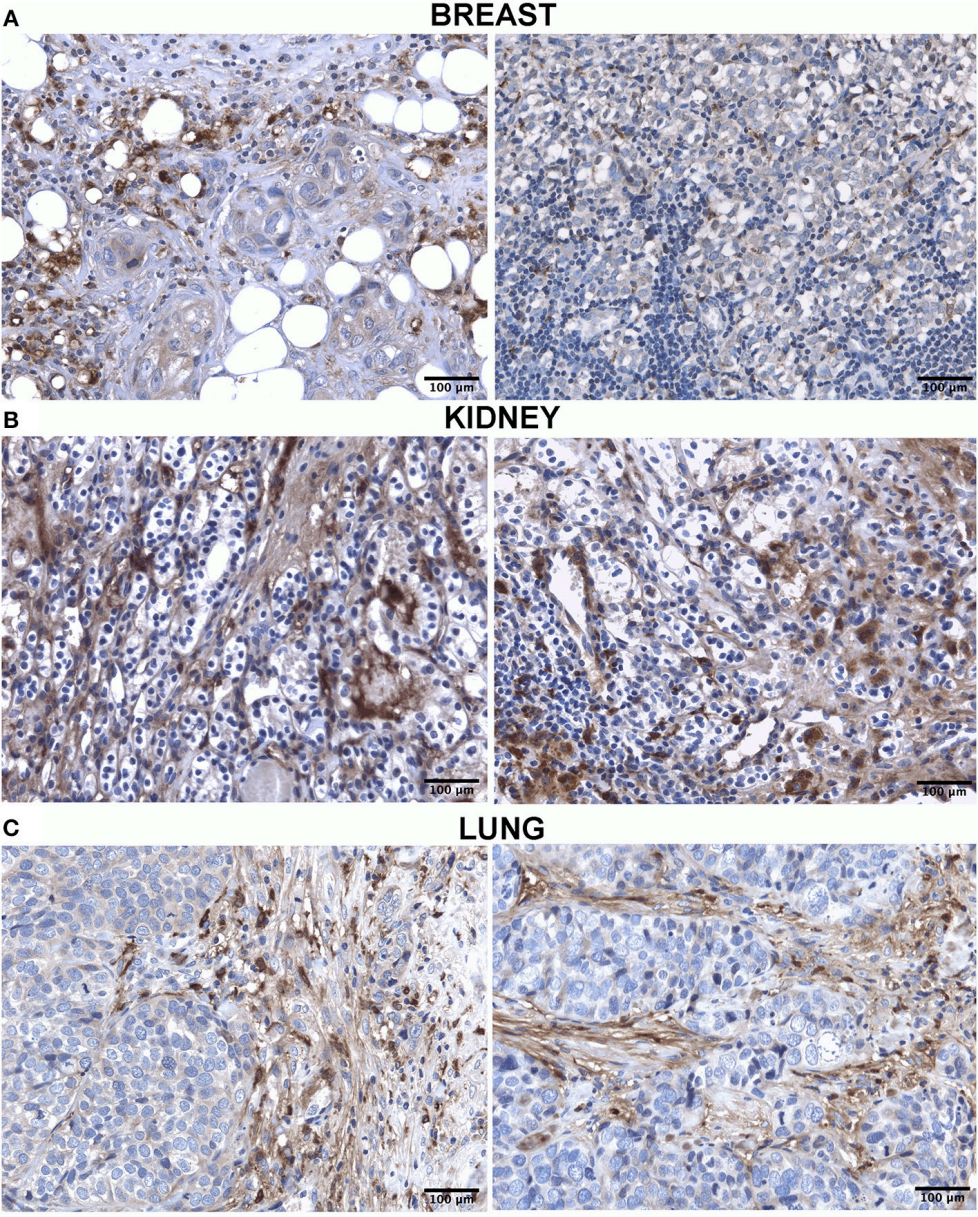
Immunohistochemistry analysis for C1q in breast **(A)**, kidney **(B)** and lung carcinoma **(C)**. Representative microphotographs showing expression of C1q in different carcinoma. The expression of C1q in carcinoma was observed in all tissues with differential distribution in the TME, as described in the result section. DAB (brown) chromogen was used to visualize the binding of anti-human C1q antibodies; scale bars, 50μm.

The IHC analysis within the BLBC microenvironment revealed that C1q was diffusely present in the tumor stroma and was expressed by macrophage-like cells, suggestive of tumor-infiltrating myeloid elements (Figure 2A).

### C1q Expression in Kidney Carcinoma has a Negative Correlation

**Figure 3:**
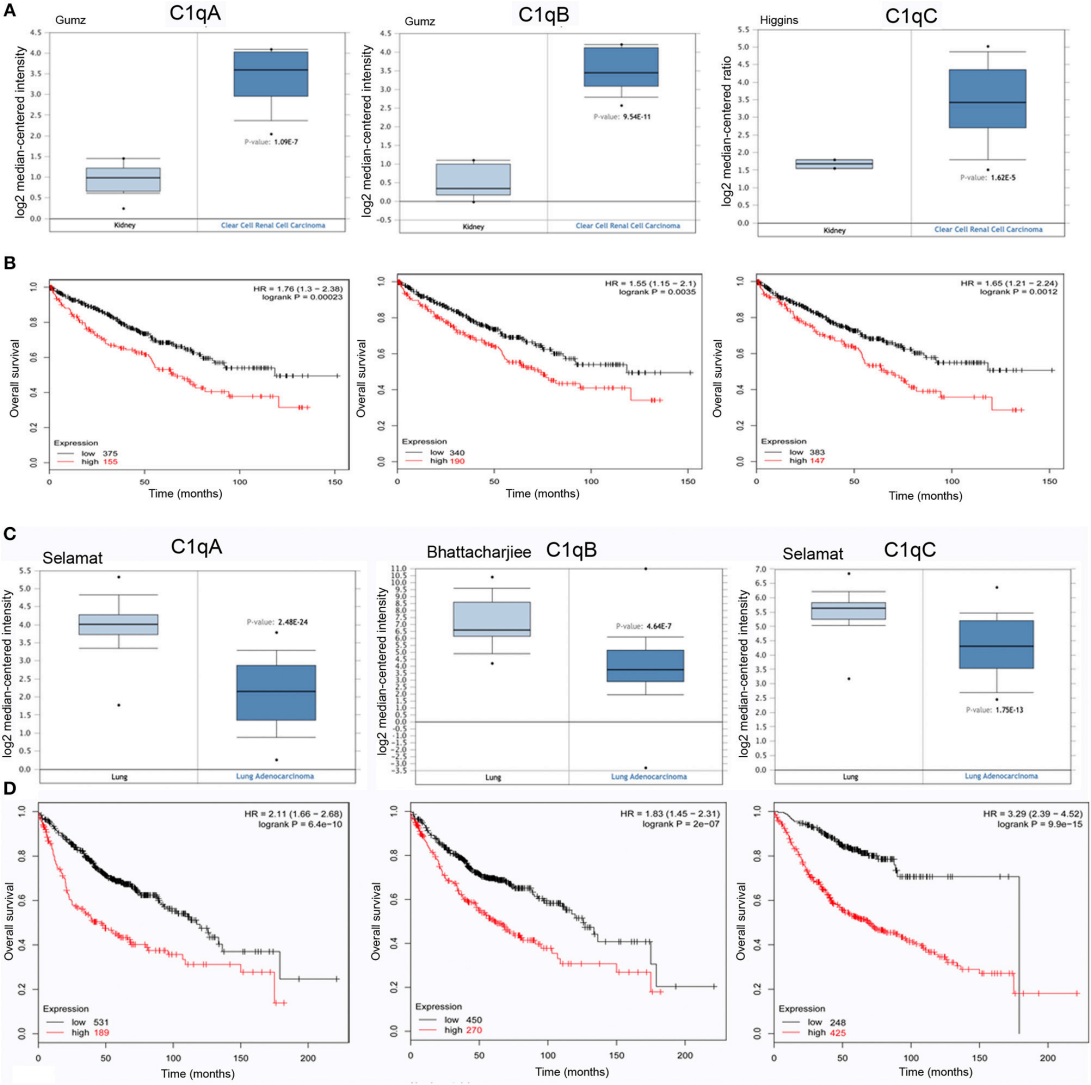
Pathological significance of C1q expression kidney and in lung cancer. Gumz's dataset have explored *C1QA* and *C1QB* mRNA expression in kidney, whereas Higgins' dataset were used for *C1QC* mRNA expression. A higher C1q expression was detectable in CCRCC cancer than that in normal tissue **(A)**, *p* < 0.05. According to the data from Kaplan-Meier plotter, C1q mRNA expressions were negatively related to an overall survival rate of the patients with CCRCC **(B)**. HR, hazard ratio. Selamat's dataset have revealed a lower *C1QA* and *C1QC* mRNA expression in lung adenocarcinoma that in normal lung tissues **(C)**, whereas Bhattacharjiee's dataset was used for *C1QB*, but the results were in accordance with Selamat's one. There was a negative association between C1q mRNA expression and a favorable prognosis in patients with lung cancer, for Kaplan-Meir plotter **(D)**. HR, hazard ratio.

The *C1QA, C1QB*, and *C1QC* mRNA expression was evaluated in kidney cancer based on the results obtained from different datasets. In CCRCC, the expression of the three C1q chains was higher as compared to normal kidney (Figure 3A, *p* < 0.05). However, in the case of PRCC, this trend was evident only for *C1QA* and *C1QB* mRNA expression (data not shown). The data obtained from Kaplan-Meier plotter showed a negative relationship between *C1QA, C1QB*, and *C1QC* mRNA expression and OS rate of patients with CCRCC (Figure 3B, *p* < 0.05). No correlation was observed between *C1QA, C1QB*, and *C1QC* mRNA expression and OS in the PRCC patients (Table 1).

Within the CCRCC microenvironment, C1q was found to be mainly expressed in the tumor stroma and in the small vessels, and it was associated with the cell membrane of tumor cells (Figure 2B).

### Lower Level of C1q Expression in Lung Carcinoma

While examing *C1QA and C1QB* mRNA expression in lung cancer, using Selamat's, Wachi's and Bhattacharjee's datasets, we found a lower expression level in adenocarcinoma (Figure 3C, *p* <0.05) and in squamous cell carcinoma (data not shown, *p* <0.05) than in normal lung tissue; *C1QC* mRNA expression was significant only in lung adenocarcinoma. As shown in Figure 3D, *C1QA, C1QB*, and *C1QC* mRNA expression levels negatively correlated with an OS rate of the patients with lung adenocarcinoma (*p* <0.05); no correlation with OS was observed in squamous cell carcinoma (Table 1).

IHC in lung adenocarcinoma revealed C1q staining in the stroma and some macrophage-like positive cells into the tumor mass (Figure 2C).

## Discussion

In this paper, we performed bioinformatics analysis to explore if C1q level could act as a possible prognostic marker in various carcinomas, in view of its reported dichotomous effects on cancer cells (pro- and anti-tumorigenic). C1q is present in colon, lung, breast, pancreatic carcinoma, and melanoma. C1q can promote adhesion, proliferation and migration of melanoma cells (17). We found C1q in abundance in all histological variants (epithelioid, sarcomatoid, and biphasic) of asbestos-induced malignant pleural mesothelioma. C1q bound high and low molecular weight HA and acted as a tumor-promoting factor (18). In addition, C1q exerted a protective effect against apoptosis, suggesting an overall pro-tumorigenic activity (17). However, Hong et al. recently observed that C1q, expressed in normal prostate, was downregulated in benign prostatic hyperplasia and prostate cancer (15). C1q was able to induce apoptosis and growth suppression of human prostate DU145 cells, through direct activation of the tumor suppressor WW-domain containing oxidoreductase (WOX1). C1q also have a pro-apoptotic effect on an ovarian cell line, SKOV3, acting via a TNF-α induced apoptosis pathway that involves upregulation of Bax and Fas (16).

In a syngeneic murine model of melanoma in C57BL/6 strain, C1q-deficient mice showed prolonged survival and slower tumor growth, as compared to wild-type mice (17). However, Bandini et al. found that neuT mice, a genetically engineered mouse model for mammary carcinoma that was made deficient for the C1qA chain (neuT-C1KO mice), manifested an accelerated tumor growth associated with an increased number of intra-tumoral vessels, compared to wild-type neuT mice. These differences in tumor progression were attributed to a reduced activation of WW domain containing oxidoreductase (WWOX) in C1q–deficient mice (25).

In view of these rather contradicting roles of C1q in tumor progression, we performed a systematic bioinformatics analysis of the expression of C1q, and its correlation with the survival rate in different carcinoma histotypes, using Oncomine and Kaplan-Meier plotter tools. We selected the carcinomas that showed all the three chains of human C1q statistically significant for the prognosis; in several cases, the prognosis was differentially linked to the C1q chains, or limited to one or two C1q chains. We often noticed the mRNA encoding for only one or two C1q chains, something that would impede synthesis of a functional C1q molecule. Indeed, we have provided evidence in the past that the expression of C1qC chain is essential for the production of functional C1q by the endothelial cells of the decidua (26). Moreover, mesothelioma cells are impaired in C1q A chain synthesis (18).

Our bioinformatics analysis highlighted that high levels of C1q have a favorable prognostic index in BLBCs for DFS and HER2^+^ breast cancer for OS, (Graphical Abstract) consistent with the *in vivo* studies by Bandini et al. using C1q-deficient mice (25). Inflammation is a major characteristic of these types of tumors. One possible explanation for the observed positive association between C1q expression and favorable prognostic index could be due to the correlation between the presence of C1q and dendritic cells (CD11c positive cells) in TME. High CD11c expression in BLBCs is associated with a significantly higher OS (*p* = 0.047) as compared to low CD11c expression (27). Dendritic cells themselves can be a potential source of C1q within the TME (28, 29). C1q, although present, is not able to bind BLBC cells (MDA-MB-231), and hence, not able to promote tumor progression (unpublished data), probably due to downregulation of putative C1q receptor(s). It is thus crucial to understand the differences in good prognosis survival between BLBCs and HER2^+^ breast cancer, the role of inflammation, and that of C1q in determining such differences.

Wilson et al. (30) found that C1q chain genes were enriched in the stroma compartment of triple-negative breast cancers. The analysis of publicly available data sets revealed that the genes encoding for the C1q chains were associated with a poor prognosis in BLBC using the TCGA dataset (504 patients). In our analysis, using the GEO dataset that include 5,143 patients, we observed a positive prognostic effect for BLBCs in DFS and HER2-positive breast cancers in OS. The opposite results were obtained for CCRCCs and lung adenocarcinomas in OS.

A negative prognostic effect arose from the analysis of kidney and lung carcinomas (Graphical Abstract). The most frequent histological subtypes include CCRCC and PRCC (CCRCC ~75%; PRCC ~10%) (31). The expression of C1q in kidney cancer is increased as compared to normal kidney tissue (Figure 3A) and C1q has a negative prognostic effect in the case of CCRCC (Figure 3B); no association was evident for PRCC. CCRCC tumor is characterized by an increased response to HIF that promotes blood vessel growth. Targeted therapies directed against VEGF, VEGF receptor, and mTOR play a crucial role in the management of metastatic CCRCC (32). We can hypothesize that C1q can also participate in promoting angiogenic processes in this particular tumor (14).

C1q has a negative prognostic value in lung tumors limited to adenocarcinomas, the most common form of lung cancer (Figure 3D). According to the WHO classification of lung tumors, there are four major histological types: adenocarcinomas, squamous cell carcinomas, large cell carcinomas, and small cell carcinomas (33). It is worth noting that C1q expression is reduced in lung cancer compared to the normal lung as we observed for surfactant protein D (SP-D) (34). Although C1q expression in lung cancer is lower than in normal tissue, lung cancer cells bind C1q present in the tumor microenvironment and activate the classical complement pathway (35). Tumor transformation is also concomitant with the loss of key defense molecules entrusted with early recognition and removal of the altered self (36).

A number of factors can modulate the role of C1q in the TME. C1q interaction with the ECM components can adversely interrupt its putative functions, as is the case with HA. It is also possible that certain tumors downregulate the putative receptor for C1q in order to escape possible apoptosis induction. Proliferative and apoptotic responses to C1q can be dictated by distinct receptors that are yet to be discovered. Last but not the least, the orientation of the C1q molecule, while engaging with the tumor cells, can also define the C1q-mediated implications. Our study encompasses all the above-mentioned possibilities, including tumor heterogeneity.

## Ethics Statement

This study was carried out as per the recommendations of governmental guidelines, and approved by the CEUR (Comitato Etico Unico Regionale, FVG, Italy; number 34/2016). All subjects gave written informed consent in accordance with the Declaration of Helsinki.

## Author Contributions

AM and RB: conception and design. CA, DB, and AR: development of methodology. BB, DB, CA, and GZ: acquisition of data. AM, DB, BB, FZ and CA: analysis and interpretation of data (e.g., statistical analysis, biostatistics, and computational analysis). RB, UK, CA, PZ, and GR: writing, review, and/or revision of the manuscript. RB: study supervision.

### Conflict of Interest Statement

The authors declare that the research was conducted in the absence of any commercial or financial relationships that could be construed as a potential conflict of interest.

## Abbreviations

TME: Tumor microenvironment
ECM: extracellular matrix
BLBC: basal-like breast cancer
CCRCC: clear cell renal cell carcinoma
PRCC: papillary renal cell carcinoma
OS: overall survival
DFS: disease-free survival
WOX1: WW-domain containing oxidoreductase.
