# Supplementary material for: Is the Complement Protein C1q a Pro- or Anti-tumorigenic Factor? Bioinformatics Analysis Involving Human Carcinomas
Source: Front Immunol. 2019 May 3;10:865. doi: 10.3389/fimmu.2019.00865 (PMC6509152; doi:10.3389/fimmu.2019.00865)
Supplement: Supplementary file 1 [file Table_1.DOCX]

| **Datasets** | **Study Description** | **Experiment Type** |
| --- | --- | --- |
| Karnoub Breast | Twenty-two (22) breast samples, including 15 non-tumor breast stroma samples and 7 primary invasive ductal breast carcinoma stroma samples, were analysed on Affymetrix U133 Plus 2.0 microarrays. | mRNA |
| Finak Breast | Fifty-three (53) breast tumor stroma samples and six (6) normal breast stroma samples were analysed on Agilent 44K microarrays. Sample data includes outcome, recurrence, grade, ER status, PR status, ERBB2 status, lymph node status, age, tumor size, and others. This data was from a dye swap study. The data in Oncomine has been processed by inversing the ratios and averaging the two values per sample. | mRNA |
| Curtis Breast | One thousand nine hundred ninety-two (1,992) breast carcinoma samples and 144 paired normal breast samples were analysed for the METABRIC project. Sample data includes ER/PR/ERBB2 status, overall survival status and follow-up time, stage, grade, and others. | mRNA |
| Perou Breast | Sixty-two (62) breast carcinoma and 3 normal breast samples were analysed on cDNA microarrays. Sample data includes type, oestrogen receptor status, grade, HER2 status, primary/metastatic, patient ID, and treatment status. | mRNA |
| Lenburg Renal | Nine (9) clear cell renal cell carcinoma and 9 normal kidney samples were analysed on Affymetrix U133A/B microarrays. Sample data includes type, age, capsule penetration, Fuhrman grade, sex, side, and sinus invasion. | mRNA |
| Gumz Renal | Ten (10) clear cell renal cell carcinoma samples, as well as ten (10) patient-matched normal tissue samples were analysed on Affymetrix U133A and U133B microarrays. | mRNA |
| Higgins Renal | Twenty-six (26) clear cell renal cell carcinoma, 5 renal cell granular carcinoma, 4 papillary renal cell carcinoma, 3 chromophobe renal cell carcinoma, 2 renal oncocytoma, 1 renal angiomyolipoma, and 3 normal kidney samples were analysed on cDNA microarrays. Sample data includes grade and type. | mRNA |
| Jones Renal | Thirty-two (32) clear cell renal cell carcinoma, 12 renal oncocytoma, 11 papillary renal cell carcinoma, 8 renal pelvis urothelial carcinoma, 6 chromophobe renal cell carcinoma, and 23 normal kidney samples were analysed. Sample data includes age, sex, grade, TNM stage, and cancer sample site. | mRNA |
| Yusenko Renal | Sixty-seven (67) various renal neoplasm samples were analysed on Affymetrix U133 Plus 2.0 microarrays. Samples include 26 conventional renal cell carcinomas, 19 papillary renal cell carcinomas, 4 chromophobe renal cell carcinomas, 4 Wilms tumors, 4 renal oncocytomas, 2 collecting duct carcinomas, 1 clear cell sarcoma of the kidney, 1 renal lipoma, 1 rhabdoid tumor of the kidney, 3 normal adult kidney, and 2 normal fetal kidney samples. | mRNA |
| Selamat Lung | Fifty-eight (58) lung adenocarcinoma and 58 normal lung (57 paired) samples were analysed. Sample data includes age; race/ethnicity; smoking status; stage; and KRAS, EGFR, and STK11 mutation status. | mRNA |
| Wachi Lung | Five (5) squamous cell lung carcinoma and 5 normal lung samples were analysed on Affymetrix U133A microarrays. Sample data includes type and patient ID. | mRNA |
| Bhattacharjee Lung | One hundred thirty-nine (139) lung adenocarcinoma, 21 squamous cell lung carcinoma, 20 lung carcinoid tumor, 6 small cell lung carcinoma, and 17 normal lung samples were analysed on Affymetrix U95A microarrays. Sample data includes type, age, M stage, max tumor percentage, N stage, primary/metastatic, recurrence, sex, site of metastasis, smoking rate (packs per year), stage, survival, and T stage. | mRNA |

**Table 1.** Characteristics of the datasets used in bioinformatics analysis with Oncomine.
