# Supplementary material for: Is the Complement Protein C1q a Pro- or Anti-tumorigenic Factor? Bioinformatics Analysis Involving Human Carcinomas
Source: Front Immunol. 2019 May 3;10:865. doi: 10.3389/fimmu.2019.00865 (PMC6509152; doi:10.3389/fimmu.2019.00865)
Supplement: Supplementary file 2 [file Table_2.docx]

| Luminal A | ER+ e/o PR+ /Her2- |
| --- | --- |
| Luminal B | ER+ e/o PR+ /Her2+ |
| Her2+/ER-/PR- | ER- /PR- /Her2+ |
| Basal-like | ER- /PR- /Her2- |

**Table 2**. Molecular classification of breast carcinoma. Breast cancers include a group of diseases with specific clinical, histopathologic and molecular properties. By transcriptome analyses of human breast cancer, they are classified in four molecular subtypes, namely luminal A, luminal B, human epidermal growth factor receptor 2 (HER2)-enriched and basal-like. These molecular subtypes have prognostic and predictive value: the HER2-overexpressing and basal-like breast cancers have poor outcomes, and within the oestrogen receptor (ER)–positive subtypes the luminal B cohort has a significantly worse prognosis than luminal A. Basal-like breast cancer (BLBCs) is a particularly aggressive molecular subtype Carcinomas with a basal-like phenotype are triple-negative (oestrogen receptor, progesterone receptor, HER2) and express at least one basal marker (CK5/6, CK14, CK15, CK17 or EGFR).
